# Atopic dermatitis increases the effect of exposure to peanut antigen in dust on peanut sensitization and likely peanut allergy

**DOI:** 10.1016/j.jaci.2014.10.007

**Published:** 2015-01

**Authors:** Helen A. Brough, Andrew H. Liu, Scott Sicherer, Kerry Makinson, Abdel Douiri, Sara J. Brown, Alick C. Stephens, W.H. Irwin McLean, Victor Turcanu, Robert A. Wood, Stacie M. Jones, Wesley Burks, Peter Dawson, Donald Stablein, Hugh Sampson, Gideon Lack

**Affiliations:** a Paediatric Allergy, Department of Asthma, Allergy and Respiratory Science, King's College London, Guys' Hospital, London, United Kingdom; b Paediatric Allergy, National Jewish Health, Denver, Colo; c Department of Pediatrics, Icahn School of Medicine at Mount Sinai, Jaffe Food Allergy Institute, New York, NY; d Centre for Dermatology and Genetic Medicine, College of Life Sciences and College of Medicine, Dentistry and Nursing, University of Dundee, Dundee, United Kingdom; e Department of Public Health Science, School of Medicine, King's College London, London, United Kingdom; f Department of Pediatrics, Division of Allergy and Immunology, Johns Hopkins University School of Medicine, Baltimore, Md; g Department of Pediatrics, University of Arkansas for Medical Sciences and Arkansas Children's Hospital, Little Rock, Ark; h Department of Pediatrics, University of North Carolina, Chapel Hill, NC; i EMMES Corporation, Rockville, Md

**Keywords:** Atopic dermatitis, peanut sensitization, peanut allergy, environmental peanut exposure, dust, AD, Atopic dermatitis, CoFAR, Consortium of Food Allergy Research, EPE, Environmental peanut exposure, FLG, Filaggrin, IQR, Interquartile range, LLQ, Lower limit of quantitation, LR, Logistic regression, OR, Odds ratio, PA, Peanut allergy, sIgE, Specific IgE, SPT, Skin prick test

## Abstract

**Background:**

History and severity of atopic dermatitis (AD) are risk factors for peanut allergy. Recent evidence suggests that children can become sensitized to food allergens through an impaired skin barrier. Household peanut consumption, which correlates strongly with peanut protein levels in household dust, is a risk factor for peanut allergy.

**Objective:**

We sought to assess whether environmental peanut exposure (EPE) is a risk for peanut sensitization and allergy and whether markers of an impaired skin barrier modify this risk.

**Methods:**

Peanut protein in household dust (in micrograms per gram) was assessed in highly atopic children (age, 3-15 months) recruited to the Consortium of Food Allergy Research Observational Study. History and severity of AD, peanut sensitization, and likely allergy (peanut-specific IgE, ≥5 kU_A_/mL) were assessed at recruitment into the Consortium of Food Allergy Research study.

**Results:**

There was an exposure-response relationship between peanut protein levels in household dust and peanut skin prick test (SPT) sensitization and likely allergy. In the final multivariate model an increase in 4 log_2_ EPE units increased the odds of peanut SPT sensitization (1.71-fold; 95% CI, 1.13- to 2.59-fold; *P* = .01) and likely peanut allergy (PA; 2.10-fold; 95% CI, 1.20- to 3.67-fold; *P* < .01). The effect of EPE on peanut SPT sensitization was augmented in children with a history of AD (OR, 1.97; 95% CI, 1.26-3.09; *P* < .01) and augmented even further in children with a history of severe AD (OR, 2.41; 95% CI, 1.30-4.47; *P* < .01); the effect of EPE on PA was also augmented in children with a history of AD (OR, 2.34; 95% CI, 1.31-4.18; *P* < .01).

**Conclusion:**

Exposure to peanut antigen in dust through an impaired skin barrier in atopically inflamed skin is a plausible route for peanut SPT sensitization and PA.

Skin barrier dysfunction plays an important role in the development of atopic dermatitis (AD),^1,2^ and AD is often cited as the first step in the allergic march.^3,4^ There is a clear association between early-onset AD and food allergy^5,6^ and a growing body of evidence that epicutaneous exposure to peanut through an impaired skin barrier increases the risk of peanut sensitization and clinically confirmed peanut allergy.^7-9^ Among children with peanut allergy with AD in the Avon Longitudinal Study of Parents and Children, 90% had been exposed to creams containing *Arachis* (peanut) oil in the first 6 months of life.^6^ In BALB/c mice epicutaneous peanut exposure has been shown to induce a potent allergic T_H_2-type response and anaphylaxis after a single oral antigen challenge^7-9^; however, in these studies this was only achieved if skin stripping, leading to skin barrier impairment and inflammation, was performed before antigen application. In flaky tail mice that carry a mutation within the murine *flg* gene, topical application of ovalbumin leads to a cellular infiltrate and antigen-specific antibody response, even without skin stripping.^10^

We have shown that early exposure to peanut antigen in household dust is a risk factor for the development of peanut sensitization and clinically confirmed peanut allergy in children who carry a filaggrin *(FLG)* null mutation in the Manchester Asthma and Allergy Study cohort.^11^ In another study environmental exposure to peanut measured indirectly based on household peanut consumption was associated with peanut allergy, particularly when compared with atopic children.^12^ Peanut protein in household dust was not objectively quantified in this study; however, other studies have measured peanut allergens in dust,^13,14^ and we have shown that peanut allergen levels in dust from the infant's bed and play area correlate with household peanut consumption and stimulate an allergic response in effector cells of patients with peanut allergy.^15^

We hypothesized that an impaired skin barrier in children with AD or *FLG* null mutations would modify the effect of environmental peanut exposure (EPE), as defined by peanut protein in household dust (in micrograms per gram), on peanut sensitization and allergy. If proved, this hypothesis would support the notion that a primary mode leading to the development of peanut sensitization and allergy occurs through presentation of environmental peanut antigen through an impaired skin barrier to underlying antigen-presenting cells. The purpose of this study was to assess whether early EPE increases the risk of peanut sensitization and allergy in young atopic children.

## Methods

Participants were from the National Institutes of Health–sponsored Consortium of Food Allergy Research (CoFAR). The design and methodology are described elsewhere.^16^ In brief, 512 children less than 15 months of age were recruited with a convincing clinical history of cow's milk allergy, egg allergy, or both and a positive skin prick test (SPT) response to cow's milk, egg, or both, respectively, or with moderate-to-severe AD with a positive SPT response to cow's milk, egg, or both but without known peanut allergy. Study procedures were reviewed and approved by a National Institute of Allergy and Infectious Diseases Data Safety Monitoring Board and by local institutional review boards, and written signed informed consent was obtained. The analyses included 359 (70.1%) of 512 participants who provided enough dust to analyze approximately 10 mg for peanut protein.

SPTs were performed with the GreerPick (Greer Laboratories, Lenoir, NC) on the infant's back. Results were obtained after 15 minutes, and the average mean wheal diameter (after subtraction of the saline negative control) was recorded. Children with peanut SPT responses of 3 mm or greater were described as peanut SPT sensitized, and children with peanut SPT responses of less than 3 mm were described as not sensitized. Children with serum specific IgE (sIgE) to peanut (ImmunoCAP system; Thermo Fisher Scientific, Uppsala, Sweden) of 0.35 kU_A_/mL or greater were described as peanut sIgE sensitized. Children with serum sIgE levels to peanut of 5 kU_A_/mL or greater were described as having a serologic diagnosis of likely peanut allergy (PA); this was postulated as in previous studies, 70% to 90% of 5- to 7-year-old children had positive diagnostic peanut challenge results with this level of peanut sIgE.^17-19^ Children were defined as not peanut allergic if they had a history of tolerating eating peanut (regardless of sensitization status) or if they were not sensitized to peanut, even if there was no history of peanut ingestion. Peanut-sensitized children (peanut SPT response ≥3 mm or peanut sIgE level of between 0.35 and 5 kU_A_/mL) without a history of peanut ingestion were excluded from the PA analysis because they did not undergo a peanut challenge at baseline and thus could not be defined as having peanut allergy or peanut tolerance. Of 359 subjects with available living room dust, 150 (41.8%) children had no history of ingestion of peanut and peanut SPT responses of 3 mm or greater or sIgE levels of 0.35 kU_A_/mL or greater and thus were excluded from the PA analysis. Of the remaining children, 89 (42.6%) of 209 were considered to have a serologic diagnosis of PA because of a peanut sIgE level of 5 kU_A_/mL or greater. There were 120 children considered not to have peanut allergy who either reported peanut consumption without a reaction (n = 20/209 [9.6%]) or who were not sensitized to peanut (n = 100/209 [47.8%]).

*FLG* genotyping was performed with genomic DNA extracted from blood. The *FLG* null mutations R501X, 2282del4, S3247X, and R2447X were assessed with a TaqMan-based allelic discrimination assay (Applied Biosystems, Life Technologies, Cheshire, United Kingdom) by using previously described probes and primers.^20,21^ History of AD and maximum severity of AD were graded by (1) extent of disease (by “rule of 9”), (2) course of disease (by history), and (3) intensity of disease (disturbance of night's sleep by itching), each on a 3-point scale, as previously described.^22^ The rule of 9 is used to calculate the area of the body's skin affected for SCORAD score assessment, where the head and neck amount to 9%, the upper limbs amount to 9% each, the lower limbs amount to 18% each, the anterior trunk amounts to 18%, the back amounts to 18%, and the genitals amount to 1%.^23^

EPE was quantified from dust collected at baseline from the family's living room floor. Families were asked to avoid vacuuming their living room floors for 3 days before obtaining dust. Participants were provided with a DUSTREAM adaptor and collector (Indoor Biotechnologies, Warminster, United Kingdom), a nylon collection filter, a disposable template, and instructions for vacuuming. The living room floor was vacuumed for 2 minutes within a 1-m^2^ surface area. Dust samples were sieved, and fine dust was extracted in a proportional volume of extraction solution.^24^ Peanut protein in dust was determined by using the Veratox polyclonal ELISA against whole peanut protein (Neogen, Lansing, Mich), which has been validated for sensitivity, specificity, and reliability in measuring peanut protein in food^25,26^ and dust.^24^ The lower limit of quantification (LLQ) of the assay was defined as 100 ng/mL whole peanut (25 ng/mL peanut protein), and samples of less than this value were defined as LLQ/2 (12.5 ng/mL peanut protein, which equated to between 1.05 and 1.23 μg/g depending on the weight of dust obtained).^27^ There were 16 (4.5%) of 359 living room dust samples with peanut protein levels of less than the LLQ. Results were converted from nanograms per milliliter into micrograms of peanut protein per gram of dust. Participant information was kept blind from the researcher performing the ELISA dust analyses. Dust samples were also obtained from the infant's bed dust; details are described in the Methods section in this article's Online Repository at www.jacionline.org.

### Statistical analysis

Data were entered into SPSS (SPSS 19.0; SPSS, Chicago, Ill) and STATA (STATA/IC 12.1; StataCorp, College Station, Tex) spreadsheets for analysis. Associations between demographic, clinical, and household factors and peanut SPT sensitization and PA were assessed by using a logistic regression (LR) model for children with available dust for analysis. Peanut protein levels in dust (micrograms per gram) underwent log_2_ transformation to normalize data. EPE spanned approximately 12 log_2_ scales (1.05-3761.68 μg/g), and therefore we showed the effect of 4 log_2_ unit increases in EPE on peanut SPT sensitization and PA. In a stepwise process all factors with a trend toward an association with peanut SPT sensitization or PA on univariate analysis (*P* < .15) were included in the multivariate model, and then only those covariates with a *P* value of less than .05 were included in the final multivariate model. The same covariates were included in the multivariate analysis for all children, children with a history of AD, and children with a history of severe AD. We assessed EPE as a continuous variable and as quartiles by dividing the span of continuous EPE into 4 equal groups. Visual graphs were inspected, and the linearity of the logit (p/[1−p]) and log_2_ continuous peanut protein level was reasonable for both peanut SPT sensitization and PA on univariate analysis. Overlapping 95% CIs of odds ratios (ORs) among EPE quartiles supported the linearity of the exposure-response relationship between log_2_ EPE and the logit of Prob (peanut SPT sensitization = positive) and Prob (PA = positive). Therefore we used continuous EPE as the optimum representation of the primary exposure variable throughout the article.

The effect of EPE on peanut sensitization or PA was assessed in a univariate and multivariate LR model in all children and subgroups of children without a history of AD, with a history of AD, or with a history of severe AD. We subsequently included an interaction term with EPE and a history of AD (vs no AD) or history of severe AD (vs no AD). To establish the relationship between EPE during the child's early life and maternal peanut consumption in pregnancy, peanut protein levels in living room dust (in micrograms per gram) were compared in homes in which mothers either avoided or consumed peanut during pregnancy by using the Mann-Whitney *U* test. Statistical significance was assessed at a *P* value of less than .05.

## Results

### EPE is associated with peanut SPT sensitization and PA

ORs (95% CIs) of factors possibly associated with peanut SPT sensitization or PA (peanut sIgE, ≥5 kU_A_/mL) are displayed in Tables I (univariate LR analysis), II, and III (multivariate LR analyses). There was a significant association between a 4-unit log_2_ increase in EPE and peanut SPT sensitization both on univariate analysis (n = 359; OR, 1.52; 95% CI, 1.08-2.14; *P* = .01) and multivariate LR analysis (n = 292; OR, 1.71; 95% CI, 1.13-2.59; *P* = .01), adjusting for parental report of hay fever ever in the child, egg SPT wheal diameter (in millimeters), and maternal peanut consumption during pregnancy and breast-feeding (which were also associated with peanut SPT sensitization at *P* < .05). There was a trend toward an association between EPE and PA on univariate analysis (n = 209; OR, 1.46; 95% CI, 0.92-2.29; *P* = .11) and a significant association on multivariate LR analysis (n = 209; OR, 2.10; 95% CI, 1.20-3.67; *P* < .01), adjusting for ethnicity, egg SPT wheal diameter, and cow's milk SPT wheal diameter (which were also associated with PA at *P* < .05). The relationship between peanut protein in the infants' bed and peanut SPT sensitization and PA is described in this article's Online Repository at www.jacionline.org.

### History of AD modifies the effect of EPE on peanut SPT sensitization and PA

**Fig 1:**
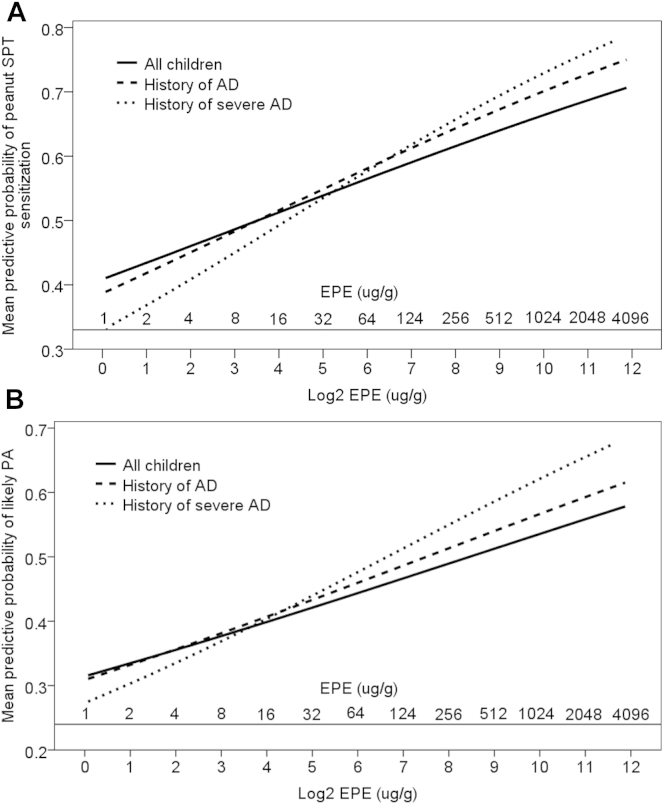
**A,** Univariate stratified predictive probability for the effect of EPE (displayed in log_2_ [microgram per gram] units and untransformed [microgram per gram] units) for peanut SPT sensitization in all children, children with a history of AD, and children with a history of severe AD. **B,** Univariate stratified predictive probability for the effect of EPE (displayed in log_2_ [microgram per gram] units and untransformed [microgram per gram] units) for likely PA in all children, children with a history of AD, and children with a history of severe AD.

On stratified univariate analysis, the effect of increasing EPE on peanut SPT sensitization and PA was augmented in children with a history of AD and severe AD (Fig 1, *A*: peanut SPT sensitization; Fig 1, *B*: PA). On univariate analysis, there was a significant interaction between EPE and AD on the risk of peanut SPT sensitization (OR, 1.41; 95% CI, 1.01-1.97; *P* < .05) per log_2_ unit EPE increase; this further increased when comparing the interaction between EPE and a history of severe AD (OR, 1.46; 95% CI, 1.04-2.07; *P* < .05). The interaction between EPE and a history of AD did not reach statistical significance for PA. There was no association between EPE and peanut SPT sensitization (OR, 0.81; 95% CI, 0.59-1.12) or PA (OR, 0.95; 95% CI, 0.60-1.49) in children without a history of AD.

**Fig E1:**
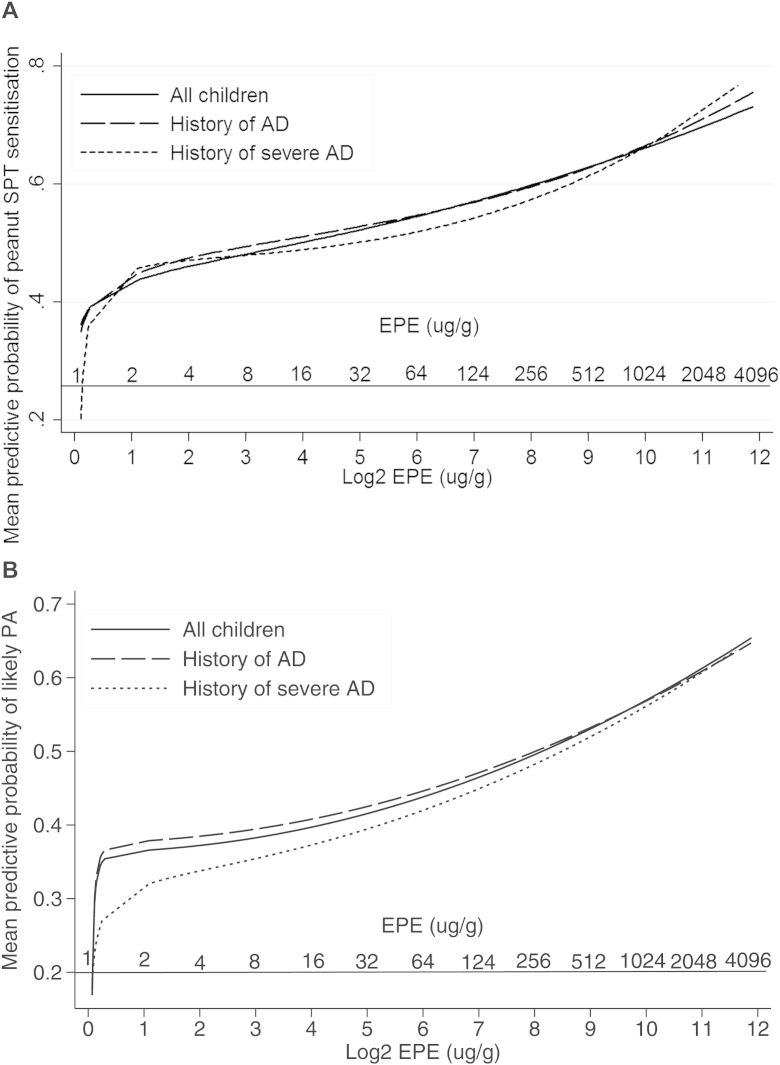
**A,** Multivariate stratified predictive probability for the effect of EPE (displayed in log_2_ [microgram per gram] units and untransformed [microgram per gram] units) for peanut SPT sensitization in all children, children with a history of AD, and children with a history of severe AD. Results are adjusted for egg SPT wheal diameter, hay fever, maternal peanut consumption during pregnancy, and breast-feeding. **B,** Multivariate stratified predictive probability for the effect of EPE (displayed in log_2_ [microgram per gram] units and untransformed [microgram per gram] units) for peanut SPT sensitization in all children, children with a history of AD, and children with a history of severe AD. Results are adjusted for egg and milk wheal diameter and ethnicity.

On multivariate LR analysis, the exposure-response relationship of EPE was augmented in children with a history of AD for peanut SPT sensitization (OR, 1.97; 95% CI, 1.26-3.09; *P* < .01) and PA (OR, 2.34; 95% CI, 1.31-4.18; *P* < .01; Table IV). For peanut SPT sensitization, the effect of EPE was further augmented in children with a history of severe AD (OR, 2.41; 95% CI, 1.30-4.47; *P* < .01); however, a similar increase was not observed for PA. In the multivariate predictive probability figures, the association between EPE and peanut SPT sensitization and PA remained; however, there was no longer a clear differentiation of the effect of EPE among all children, children with a history of AD, and children with a history of severe AD (see Fig E1, *A*, in this article's Online Repository at www.jacionline.org: peanut SPT sensitization; see Fig E1, *B*: PA).

The interaction between EPE and a history of AD for the risk of peanut SPT sensitization remained significant in the multivariate model; the OR was 1.48 (95% CI, 1.01-2.17; *P* < .05) per log_2_ unit EPE increase in children with a history of AD versus those with no AD, and the OR was 1.56 (95% CI, 1.04-2.34, *P* = .03) in children with a history of severe AD versus those with no AD. In the final multivariate model there was a trend toward an interaction between EPE and a history of AD for PA with an OR of 1.68 (95% CI, 0.91-3.12; *P* = 0.10) and an OR of 1.68 (95% CI, 0.85-3.31; *P* = .14) in children with a history of severe AD versus those with no AD.

### *FLG* genotype on peanut sensitization and PA

The prevalence of *FLG* null mutations in white children with AD (with dust available) was 14.9% (41/275); of these children, 37 had *FLG* heterozygote mutations, 3 had a combined heterozygote mutations, and 1 had a 2282del4 homozygous mutation. There was no significant association between *FLG* heterozygous or compound heterozygous/homozygous mutations and peanut SPT sensitization/PA; there was also no interaction between *FLG* genotype and EPE.

### Comparisons of the included group (n = 359) with available living room dust and the excluded group (n = 153)

There was no difference in the rate of peanut sensitization or PA between subjects with (n = 359) versus those without (n = 153) available dust; however, there were small but significant differences in the rate of severe AD, ethnicity, number of older siblings, maternal history of AD, maternal peanut consumption during breast-feeding, and peanut present in the home while breast-feeding (see Table E1 in this article's Online Repository at www.jacionline.org).

## Discussion

In this high-risk atopic cohort we found that EPE, as assessed by log_2_ transformed peanut protein (in micrograms) per gram of living room dust was a risk factor for peanut SPT sensitization and PA (peanut sIgE, ≥5 kU_A_/mL). After adjustment, an increase in 4 log_2_ EPE units increased the odds of peanut SPT sensitization 1.71-fold (95% CI, 1.13- to 2.59-fold) and the odds of PA 2.10-fold (95% CI, 1.20- to 3.67-fold). The effect of EPE on peanut SPT sensitization and PA increased in an exposure-dependent manner in children with a history of AD, with an increase in odds of 1.97 and 2.34, respectively. The effect of EPE on peanut SPT sensitization was further augmented in children with a history of severe AD; however, this was not the case for PA, which might be due to the smaller sample size of this group. There was a significant interaction between EPE and the history and severity of AD for peanut SPT sensitization, with a trend toward an AD-EPE interaction for PA. Given that peanut sensitization and allergy are more common in children with a history of AD,^5,6^ these data suggest that environmental exposure to peanut through an impaired skin barrier is a plausible route for peanut sensitization and allergy. The relationship between peanut protein in the infants' bed and peanut SPT sensitization and PA is discussed in this article's Online Repository at www.jacionline.org.

The egg-induced SPT wheal diameter was also associated with peanut SPT sensitization and PA. Egg allergy is known to be a strong predictor of peanut sensitization and allergy.^28^ The cow's milk–induced SPT wheal diameter was also associated with peanut SPT sensitization and PA; however, it lost significance on multivariate analysis for peanut SPT sensitization, and this might just be another marker of atopy. Environmental exposure to peanut was not a risk factor for egg SPT sensitization or milk SPT sensitization, confirming the specificity of environmental peanut levels on peanut sensitization rather than food sensitization in general. Nonwhite ethnicity (black, Asian, and other nonwhite races combined) was associated with a peanut sIgE level of 5 kU_A_/L or greater but not peanut SPT sensitization. This supports the findings of the Learning Early About Peanut (LEAP) study, in which black race was associated with a higher peanut sIgE level but a lower peanut SPT response in the baseline screening data from the LEAP study.^28^

*FLG* null mutations were not associated with peanut sensitization or PA. This differs from previous published findings; children with 1 of more *FLG* null mutations were found to have an increased risk of challenge-proven peanut allergy in white individuals from 4 different populations (United Kingdom, Irish, Dutch, and Canadian).^29^ The lack of association with *FLG* genotype might be because in CoFAR children already had a 92.5% history of AD and a 54.3% history of severe AD; thus the skin barrier was already impaired, irrespective of whether children had *FLG* null mutations. In addition, the rate of *FLG* null mutations was surprisingly low in this cohort (14.9%) given the high rate and severity of AD; previous studies have shown that *FLG* null mutations are present in up to 56% of children with moderate-to-severe AD.^21,30^ This might reflect a more varied genetic background in the white American population.^31^ Another potential explanation for the low *FLG* mutation rate in this cohort is that 104 children with known PA or peanut sIgE >5 kU_A_/L were excluded from the CoFAR study before enrollment. If these children had been included, we would have expected a higher rate of *FLG* null mutations, given the known association between peanut allergy and *FLG* null mutations.^29^ A further explanation could be that in children with cow's milk and egg allergy (one of the inclusion criteria for the CoFAR observational cohort), exposure to cow's milk or egg allergens through breast milk or small quantities in food might have led to more severe AD.

Previously, the CoFAR study showed that frequent (≥2 times weekly) maternal peanut consumption during the last trimester of pregnancy was a risk factor for a peanut sIgE level of 5 kU_A_/L or greater (OR, 2.9; 95% CI, 1.7-4.9).^32^ In the subgroup of children with available dust samples (n = 359), maternal peanut consumption during pregnancy (any trimester) was associated with peanut SPT sensitization (adjusted OR, 2.66; 95% CI, 1.18-5.99) but not with a peanut sIgE level of 5 kU_A_/L or greater (*P* > .3); frequent maternal peanut consumption (≥2 times weekly) in the last trimester showed only a trend toward an association with a peanut sIgE level of 5 kU_A_/L or greater (*P* = .15). The lack of significance for this might be due to the smaller sample size of children with available dust; however, maternal peanut consumption during pregnancy might simply be an indirect marker of EPE. Levels of peanut protein in living room dust were significantly greater in households in which mothers consumed peanuts during pregnancy (median, 45.2 μg/g; interquartile range [IQR], 17.5-161.8 μg/g) versus households in which mothers avoided peanuts during pregnancy (median, 16.6 μg/g; IQR, 4.3-72.2 μg/g; *P* = .001). A prospective study would be required in which maternal peanut consumption during pregnancy was controlled and household peanut consumption was subsequently compared with peanut protein in household dust throughout early childhood to tease out the effect of maternal peanut consumption during pregnancy and EPE during infancy.

**Fig E2:**
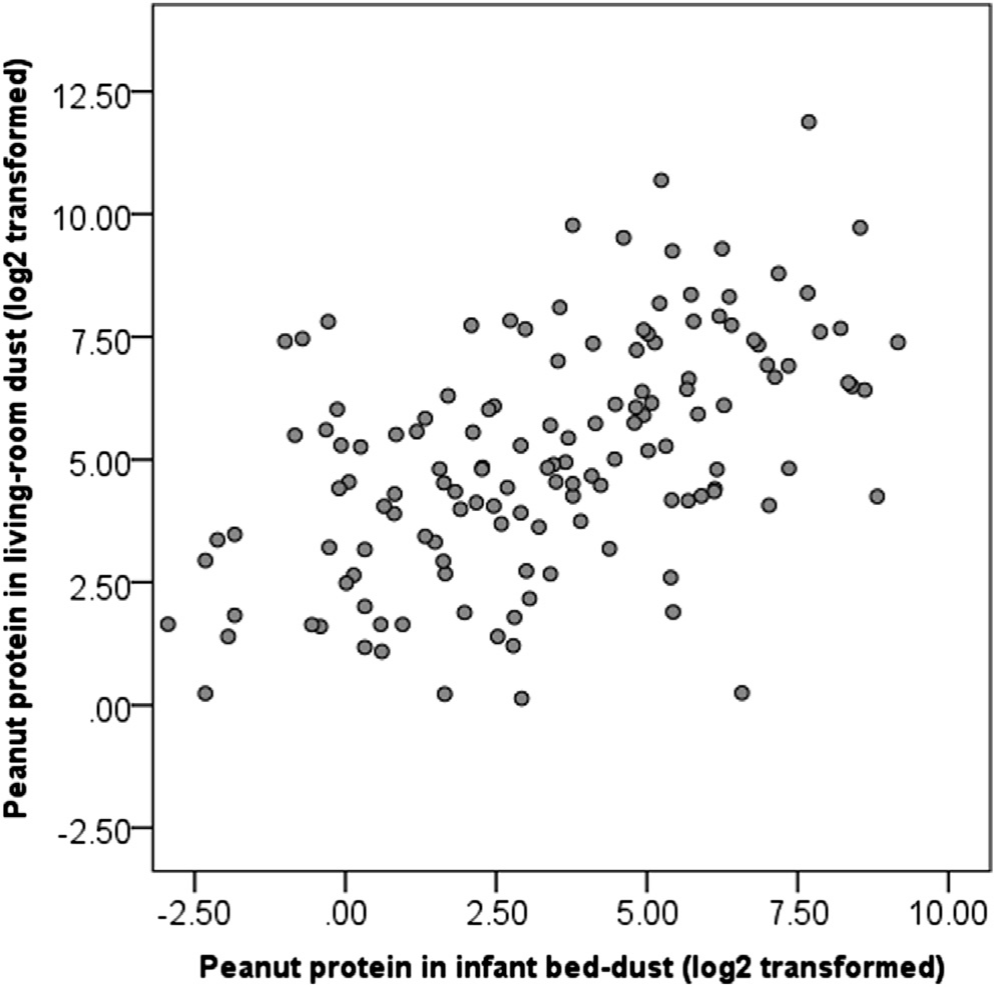
Scatter plot of peanut protein concentration in bed versus living room dust. The Spearman tank correlation coefficient *(r*_*s*_*)* was 0.521 (*P* < .001).

The limitations of this study included missing living room dust samples in 153 (30%) of 512 participants, which might have introduced an element of bias. A serologic diagnosis of PA (sIgE, ≥5 kU_A_/L) rather than one based on oral food challenges was used, which meant that 150 children were excluded because of uncertainty about their peanut allergy outcome; this could also have introduced bias. Children with known peanut allergy were excluded at baseline; these children might have had even higher peanut protein levels in living room dust and thus even steeper predictive probability curves for peanut sensitization and PA. Subjects recruited who did not have moderate-to-severe AD had either cow's milk or egg allergy; this might have led to an unusual association between EPE and peanut SPT sensitization or PA in children with no history of AD. The dust sample obtained was a single baseline collection from one area of the home and thus might be prone to variation; however, previous studies have shown high within-home correlation of peanut protein levels in dust, and peanut protein levels from a single dust collection have been shown to correlate strongly with household peanut consumption over the previous 6-month period.^15^ Peanut protein levels in dust from the living-room floor were positively correlated with those found in the infants' bed (see Fig E2 in this article's Online Repository at www.jacionline.org). There was no detailed assessment of infant peanut consumption, which could potentially protect a child from high EPE, as per the findings of Fox et al,^12^ who showed that children who consumed peanut in the first year of life were not affected by high household peanut consumption. Animal data suggest that oral allergen exposure prevents induction of allergy,^33^ whereas epicutaneous exposure prevents induction of oral tolerance.^8^ The role of early high-dose peanut consumption on the prevention of peanut allergy is currently being investigated^28^ but has already been suggested in cross-sectional observational studies.^34^

In summary, these findings demonstrate a positive association between exposure to peanut protein in dust and peanut SPT sensitization and PA in atopic children. The effect of EPE on peanut sensitization and PA was augmented in children with a history of AD and severe AD for peanut sensitization after adjusting for other covariates. This provides biological plausibility that EPE might be sensitizing children through an impaired skin barrier, thus supporting the hypothesis of epicutaneous sensitization. We demonstrated the specificity of EPE on peanut SPT sensitization and PA by showing that EPE does not increase the risk of egg or cow's milk SPT sensitization; however, it would be interesting to assess the effect of other food allergens in dust and respective sensitization and allergy to these foods. Routes of exposure to food antigens appear to be crucial in determining whether food allergy or tolerance develops as per the dual-allergen exposure hypothesis.^35,36^ Although early consumption of food will inevitably lead to higher environmental exposure to foods, there are currently studies in place assessing the role of oral tolerance induction in young children (www.leapstudy.co.uk and www.eatstudy.co.uk); should these strategies fail to prevent the development of food sensitization and allergy, the alternative strategy of reducing environmental exposure to food allergens could be considered.

**Key messages**

• Increased environmental exposure to peanut protein is associated with an increased risk of sensitization and likely allergy to peanut in atopic children.
• The effect of peanut dust exposure on peanut sensitization is augmented in children with a history of and increasing severity of AD.
• The data are consistent with the hypothesis that allergic sensitization to peanut occurs through an impaired and inflamed skin barrier.

## Figures and Tables

**Table E1:** CoFAR demographics from the included group (n = 359) with available living room dust versus the excluded group (n = 153) and whole cohort (n = 512)∗

	Included group	Variable included group	Percentage of variable included group	Excluded group	Variable excluded group	Percentage of variable excluded group	*P* value	Whole CoFAR cohort	Variable whole cohort	Percentage of variable in cohort
Peanut SPT sensitization ≥3 mm	359	196	54.6%	153	80	52.3%	.38	512	276	53.9%
Peanut sIgE ≥0.35 kU/mL	359	214	59.6%	153	91	59.5%	.96	512	305	59.6%
Likely PA (sIgE ≥5 kU/ml)	209	89	42.6%	99	45	45.5%	.40	308	134	43.5%
History of infantile AD	359	332	92.5%	153	139	90.8%	.28	512	471	92.0%
Maximum AD severity before study entry										
No AD (0)	359	27	7.5%	153	14	9.2%	.28	512	41	8.0%
Mild (3-4)	359	35	9.7%	153	17	11.1%	.41	512	52	10.2%
Moderate (5-6)	359	102	28.4%	153	47	30.7%	.34	512	149	29.1%
Severe (7-9)	359	195	54.3%	153	75	49.0%	**.04**	512	270	52.7%
Nonwhite ethnicity	359	81	22.6%	153	51	33.3%	**<.01**	512	380	74.2%
*FLG* null mutation (excluding nonwhite subjects)	275	41	14.9%	101	15	14.9%	.98	376	56	14.9%
Parental report of hay fever ever in the child	353	31	8.8%	148	11	7.4%	.33	501	42	8.4%
Male sex	357	240	67.2%	153	103	67.3%	.97	512	345	67.4%
Maternal history of atopy or asthma	356	243	68.3%	153	102	66.7%	.52	509	345	67.8%
Paternal history of atopy or asthma	350	217	62.0%	150	89	59.3%	.31	500	306	61.2%
Maternal history of AD	245	87	35.5%	97	28	28.9%	**.02**	342	115	33.6%
Paternal history of AD	215	71	33.0%	85	27	31.8%	.69	300	98	32.7%
Peanut consumption during pregnancy	352	295	83.8%	152	124	81.6%	.28	504	419	83.1%
Peanut consumption during breast-feeding	299	179	59.9%	133	64	48.1%	**<.01**	432	243	56.3%
Peanut present in house while breast-feeding	358	271	75.7%	153	107	69.9%	**.02**	511	378	74.0%
Older siblings	359	148	41.2%	153	54	35.3%	**.02**	512	202	39.5%
Egg SPT wheal diameter, median (IQR)	358	7.0 (4.0-10.5)		153	7.5 (4.3-11.0)		.32	511	7.5 (4.0-10.5)	
Cow's milk SPT wheal diameter, median (IQR)	356	5.0 (0.0-9.0)		155	4.0 (0.0-9.0)		.69	511	5.0 (0.0-9.0)	
Breast-feeding duration (mo), median (IQR)	359	5.0 (1.0-9.0)		153	5.0 (2.0-9.0)		.15	512	5.0 (2.0-9.0)	
Maternal age at baseline (y), median (IQR)	357	32.0 (28.0-35.0)		153	32.0 (28.5-35.0)		.78	510	32.0 (28.0-35.0)	
Child's age at baseline assessment (mo), median (IQR)	359	9.0 (7.0-12.0)		153	9.0 (7.0-12.0)		.64	512	9 (7.0-12.0)	
EPE (μg/g) in living room dust	359	39.1 (13.4-150.6)		153	Not available			359	39.1 (13.4-150.6)	
EPE (μg/g) in infant bed dust	172	10.7 (2.5-42.8)		340	Not available			172	10.7 (2.5-42.8)	

Statistically significant values (*P* < .05) are shown in boldface.

∗ Numbers and percentages of count data or medians (IQRs) of continuous factors and EPE values are shown.

**Table I:** Unadjusted ORs and 95% CIs measuring associations between peanut SPT sensitization and likely PA and log_2_ EPE units and subject demographic, clinical, and household factors∗

	Peanut SPT sensitization (n = 359 [54.6% positive])			Likely PA (n = 209 [42.6% positive])		
	OR	95% CI	*P* value	OR	95% CI	*P* value
4 log_2_ EPE (μg/g)†	1.52	1.08-2.14	**.01**	1.46	0.92-2.29	.11
History of infantile AD	1.83	0.82-4.06	.14	1.87	0.63-5.51	.26
Maximum AD severity before entry						
No AD (0)	Reference category			Reference category		
Mild (3-4)	2.46	0.88-6.89	.09	1.31	0.31-5.53	.71
Moderate (5-6)	1.77	0.75-4.19	.19	1.91	0.60-6.08	.28
Severe (7-9)	1.77	0.78-4.01	.17	1.94	0.64-5.88	.24
Nonwhite ethnicity	1.73	0.44-2.21	.23	1.93	1.04-3.60	**.04**
*FLG* null mutation (excluding nonwhite subjects)	0.72	0.37-1.39	.32	1.29	0.54-3.08	.56
Parental report of hay fever ever in the child	3.07	1.28-7.32	**.01**	1.52	0.57-4.20	.39
Male sex	0.82	0.53-1.28	.38	1.49	0.81-2.73	.20
Maternal history of atopy or asthma	1.29	0.83-2.02	.26	1.26	0.69-2.28	.45
Paternal history of atopy or asthma	1.00	0.65-1.54	1.0	1.00	0.56-1.78	1.0
Maternal history of AD	0.94	0.56-1.59	.82	1.13	0.56-2.28	.73
Paternal history of AD	0.74	0.42-1.30	.29	0.57	0.25-1.28	.17
Peanut consumption in pregnancy	1.67	0.94-2.26	.08	1.49	0.65-3.40	.34
Peanut consumption while breast-feeding	0.69	0.43-1.10	.12	0.51	0.27-0.94	.03
Peanut butter in house while breast-feeding	1.04	0.64-1.69	.88	0.94	0.49-1.78	.84
Older siblings	1.34	0.87-2.04	.18	1.11	0.63-1.95	.73
Egg SPT wheal diameter (mm)	1.15	1.10-1.21	**<.01**	1.26	1.17-1.35	**<.01**
Cow's milk SPT wheal diameter (mm)	1.07	1.03-1.11	**<.01**	1.21	1.09-1.35	**<.01**
Duration of breast-feeding (mo)	1.05	1.00-1.10	.08	1.12	1.04-1.20	**<.01**
Maternal age at baseline (y)	1.02	0.99-1.07	.12	1.02	0.97-1.08	.40
Child's age at baseline assessment (mo)	1.11	1.04-1.19	**<.01**	1.04	0.96-1.14	.34

Statistically significant values (*P* < .05) are shown in boldface.

∗ Descriptive statistics of subject factors and EPE are shown in Table E1.

† ORs for EPE reflect a 4-unit increase in log_2_ EPE. ORs for other continuous factors reflect a 1-unit increase in the factor unit. These include egg SPT wheal diameter, cow's milk, duration of breast-feeding, maternal age at baseline, and child's age at baseline. ORs for AD severity compare each severity level with the level “no AD.” All other factors are dichotomous. ORs compare yes with no, ever with never, or male with female.

**Table II:** Adjusted peanut sensitization (OR [95% CI]) measuring associations between EPE and subject factors (n = 292)∗

	OR	95% CI	*P* value
4 log_2_ EPE (μg/g)	1.71	1.13-2.59	**.01**
Egg SPT wheal diameter (mm)	1.17	1.11-1.24	**<.001**
Maternal peanut consumption in pregnancy	2.77	1.24-6.20	**.01**
Maternal peanut consumption while breast-feeding	0.46	0.25-0.85	**.01**
Parental report of hay fever ever in the child	3.88	1.35-11.15	**.01**

Subject factors and EPE values are significant at the 5% level (in boldface). The OR of EPE represents an increase of 4 log_2_ EPE units (in micrograms per gram).

∗ Sample size was reduced from 359 to 292 because of missing data for some factors in the multivariate analysis.

**Table III:** Adjusted likely PA (OR [95% CI]) measuring associations between EPE and subject factors (n = 209)∗

	OR	95% CI	*P* value
4 log_2_ EPE (μg/g)	2.10	1.20-3.67	**<.01**
Egg SPT wheal diameter (mm)	1.25	1.15-1.36	**<.001**
Nonwhite ethnicity	2.59	1.21-5.58	**.02**
Cow's milk SPT wheal diameter (mm)	1.14	1.06-1.22	**<.001**

Subject factors and EPE values are significant at the 5% level (in boldface). The OR of EPE represents an increase of 4 log_2_ EPE units (in micrograms per gram).

∗ The sample size was reduced from 359 to 209 because of missing data for some factors in the multivariate analysis.

**Table IV:** Stratified LR analysis of the effect of 4 log_2_ EPE units on peanut SPT sensitization and PA in all children, children with a history of AD, and children with a history of severe AD

	All participants			Participants with history of AD			Participants with history of severe AD		
	No‡	OR (95% CI)	*P* value	No.	OR (95% CI)	*P* value	No.	OR (95% CI)	*P* value
Peanut SPT sensitization∗	292	1.71 (1.13-2.59)	**.01**	269	1.97 (1.26-3.09)	**<.01**	158	2.41 (1.30-4.47)	**<.01**
Likely PA†	209	2.10 (1.20-3.67)	**<.01**	192	2.34 (1.31-4.18)	**<.01**	114	2.05 (0.98-4.29)	.06

Subject factors and EPE values are significant at the 5% level (in boldface).

∗ Adjusted for parental report of hay ever in the child, egg SPT wheal diameter, maternal peanut consumption during pregnancy, and breast-feeding.

† Adjusted for ethnicity, egg, and milk SPT wheal diameter.

‡ The sample size was reduced from 359 to 292 (peanut SPT sensitization) and 209 (likely PA) because of missing data for some factors in the multivariate analysis.

## Methods

EPE was also quantified from dust collected at baseline from the infant's bed. Families were asked to avoid changing their infant's bed sheet for 3 days before obtaining dust. Peanut protein levels in bed dust versus living room dust (in micrograms per gram) were compared by using the Mann-Whitney *U* test and the Spearman rank correlation coefficient *(r*_*s*_*)*; additionally, Pearson correlation was used to compare log_2_ transformed peanut protein levels (in micrograms per gram) in dust from the infant's bed versus living room dust.

## Results

There were only 173 (33.8%) of 512 infant bed dust samples available for peanut protein analysis in the CoFAR observational study. This was because of a large proportion of homes in which no or less than 5 mg of dust was obtained from the infant's bed. Median peanut protein concentrations in the infant's bed (10.7 μg/g; IQR, 2.5-42.38 μg/g) were also significantly lower than peanut protein levels obtained from living room dust, where the concentration was approximately 4 times higher (39.1 μg/g; IQR, 13.4-150.60 μg/g; *P* < .001). However, peanut protein levels in the infant's bed and living room floor were positively correlated (n = 138; r_s_ = 0.52; 95% CI, 0.39-0.63; *P* < .001; see Fig E2).

There was a trend toward a significant association between bed dust EPE values and peanut SPT sensitization on univariate analysis (n = 172; OR, 1.08; 95% CI, 0.97-1.21; *P* = .16); however, this was lost on multivariate analysis (n = 132; OR, 1.05; 95% CI, 0.92-1.20; *P* = .49) adjusting for ethnicity, egg SPT wheal diameter, and cow's milk SPT wheal diameter. There was no significant association between infant bed dust EPE and PA on univariate LR analysis for EPE (n = 109; OR, 0.93; 95% CI%, 0.80-1.06; *P* = .27). There was no interaction between AD or AD severity and bed dust EPE on peanut SPT sensitization or likely allergy.

## Discussion

The lack of association between peanut protein levels in infant bed dust and peanut sensitization/PA is not surprising because peanut protein levels in bed dust correlate best with individual peanut consumption^E1^ and most infants recruited to CoFAR would not have been eating peanut at this stage. This is reflected in the lower median peanut protein concentration found in the infant's bed compared with that in living room dust. The living room is the area that reflects the passage of most members of the family and thus the contribution of household peanut consumption to EPE. Furthermore, with the low number of dust samples and complete data sets we had using infant bed dust samples, we were underpowered to show an effect.
